# Weaving a 2D net of hydrogen and halogen bonds: cocrystal of a pyrazolium bromide with tetra­fluoro­diiodo­benzene

**DOI:** 10.1107/S2053229622004648

**Published:** 2022-05-09

**Authors:** Steven van Terwingen, Ben Ebel, Ruimin Wang, Ulli Englert

**Affiliations:** a Institute of Inorganic Chemistry, RWTH Aachen University, Landoltweg 1, 52074 Aachen, Germany

**Keywords:** crystal engineering, halogen bonds, hydrogen bonds, QTAIM, electron density

## Abstract

Hydro­halides of Lewis bases may act as halogen bond (XB) acceptors and combine two directional inter­actions, namely, hydrogen bonds (HB) and XBs in the same solid. 3-(1,3,5-Trimethyl-1*H*-pyrazol-4-yl)acetyl­acetone (C_11_H_16_N_2_O_2_, HacacMePz) was protonated with H*X* (*X* = Cl or Br) to afford the hydro­halides, C_11_H_17_N_2_O_2_^+^·*X* ^−^ or H_2_acacMePz^+^·*X* ^−^ (**1**, *X* = Cl; **2**, *X* = Br). Hydro­halides **1** and **2** are isomorphous and adopt a classical dipole packing. Consistent with the observation for most β-diketones, the enol form with an intra­molecular HB is observed. Additional noteworthy inter­actions are HBs of the protonated pyrazolium towards the *X* ^−^ anion at donor–acceptor distances of 2.9671 (17) Å for **1** and 3.159 (4) Å for **2**. Cocrystallization of hydro­bromide **2** with the XB donor tetra­fluoro­diiodo­benzene (TFDIB) leads to the adduct C_11_H_17_N_2_O_2_^+^·Br^−^·0.5C_6_F_4_I_2_·H_2_O or (H_2_acacMePz^+^·Br^−^)_2_·(H_2_O)_2_·TFDIB (**3**), in which the XB donor TFDIB is situated on a crystallographic centre of inversion. Classical HBs link organic cations, water mol­ecules and Br^−^ anions into chains along [010]. Almost orthogonal to this inter­action, XBs with Br⋯I = 3.2956 (4) Å connect neighbouring chains along [102] into two-dimensional sheets in the (10  ) plane. Assisted by their negative charge, halide anions represent particularly good nucleophiles towards XB donors.

## Abstract 2

In the cocrystal of the hydro­bromide of a substituted pyrazole with tetra­fluoro­diido­benzene, the bromide anion engages in both hydrogen and halogen bonds.

## Introduction

In recent decades, the field of crystal engineering has evolved rapidly (Desiraju, 2010 ▸; Aakeröy *et al.*, 2010 ▸). The ability to tailor solids for specific applications, such as gas separation (Wu *et al.*, 2018 ▸; Gao *et al.*, 2020 ▸) and storage (Müller *et al.*, 2017 ▸), sensing (Lustig *et al.*, 2017 ▸), catalysis (Rimer *et al.*, 2018 ▸) and other areas (Blagden *et al.*, 2007 ▸; Zhang *et al.*, 2018 ▸), has contributed to this triumph.

Our group has used heterobifunctional mol­ecules such as *N*-donor functionalized acetyl­acetones (Kremer & Englert, 2018 ▸) as ligands for the selective construction of heterobimetallic coordination polymers. These examples rely on covalent and coordinative bonds, sometimes in combination with hydrogen bonds, *e.g.* in metal–organic frameworks like MOF-5 (Li *et al.*, 1999 ▸). In this contribution, we focus on the weaker yet also decisive combination of two essentially electrostatic and highly directional inter­actions, hydrogen bonds (HB) and halogen bonds (XB) (Saha *et al.*, 2005 ▸; Aakeröy *et al.*, 2013 ▸). These attractive inter­actions have gained increasing inter­est in recent years from both experimental and theoretical aspects (Costa, 2018 ▸; Cavallo *et al.*, 2016 ▸). Halogen bonds are formed between a Lewis base and a (mostly heavy) halogen. The latter exhibits an electron-deficient site opposite to its σ-bond, the so-called σ-hole (Politzer *et al.*, 2007 ▸; Clark *et al.*, 2007 ▸; Politzer *et al.*, 2017 ▸). This σ-hole is particularly pronounced in polyfluorinated iodo­benzenes, in which the polarizable iodine acts as the halogen-bond donor. Lewis bases such as halides or organic mol­ecules with a lone-pair donor act as matching counterparts, *i.e.* XB acceptors. X-ray diffraction has provided information beyond geometry and confirmed the σ-hole model from theory: experimental charge–density studies based on high-resolution diffraction data have provided insight into the nature of strong (Bianchi *et al.*, 2003 ▸, 2004 ▸; Wang *et al.*, 2012 ▸, 2018*a* ▸, 2019 ▸) and weak (Otte *et al.*, 2021 ▸) XBs, and even for hypervalent iodine com­pounds, such as Togni reagent I (Wang *et al.*, 2018*b* ▸). The above-mentioned linkers in crystal engineering, our ditopic mol­ecules, not only act as Lewis bases towards metal cations but can also engage in halogen bonds as nucleophiles (Merkens *et al.*, 2013 ▸) and as Brønsted bases towards mineral acids. We recently reported the cocrystal of a substituted pyrazolium chloride and 1,2,4,5-tetra­fluoro-3,6-di­iodo­benzene (TFDIB), in which the chloride anion engages in a hydrogen and a halogen bond in an orthogonal fashion (van Terwingen *et al.*, 2021*a* ▸). Inter­estingly, we found that the reported mol­ecule does not cocrystallize with TFDIB alone, most probably due to steric hindrance around the N-donor atom. Quite obviously, a proton is much smaller than any halogen-bond donor; therefore, we exploited a hydro­halic acid to introduce this proton and a halide as a halogen-bond acceptor at the same time. The structural results were used for a single-point calculation, and the topology of the resulting electron density was analyzed by Bader’s Quantum Theory of Atoms in Mol­ecules (QTAIM) (Bader, 1990 ▸). Both hydrogen and halogen bonds are reflected in bond paths with appreciable electron density in their bond critical points (bcps). We proposed this to be prototypic for a new class of cocrystals in which hydro­halides of organic Lewis bases and halogen-bond donors co-exist in the same solid and possibly build extended structures. Further studies on our heterobifunctional mol­ecules led to the target structure of this contribution, with a substituted pyrazolium bromide, water and TFDIB as constituents. Chemical diagrams for the pyrazolium halides and the bromide cocrystal with TFDIB are shown in Scheme 1. 
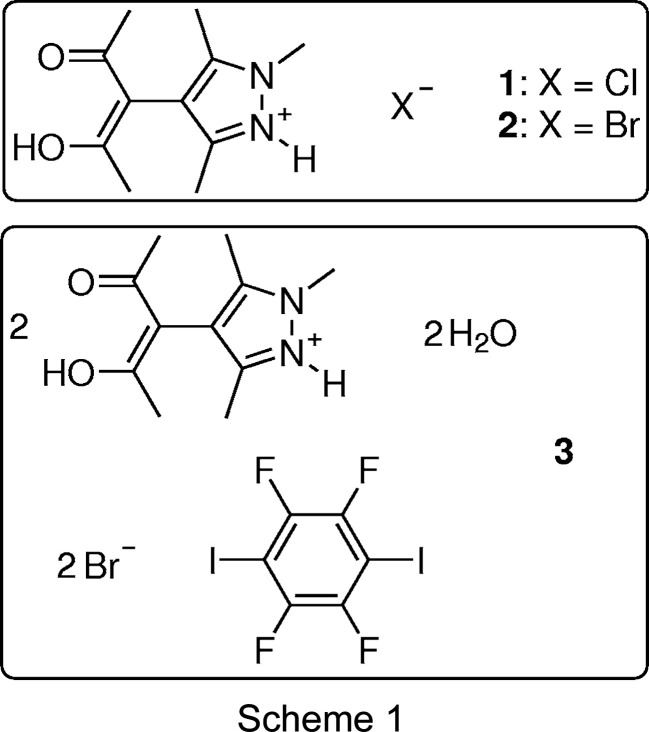


## Experimental

### Materials and methods

All chemicals were used without further purification. HacacMePz was prepared as described previously (van Terwingen *et al.*, 2021*b* ▸). Magnetic resonance spectra were measured using a Bruker Avance II Ultrashield 11 plus 400 spectrometer (400 MHz, referenced to tetra­methyl­silane). IR spectra were recorded with a Shimadzu IRSpirit IR spectrometer using the ATR–IR method (ATR is attenuated total reflectance). Elemental analyses were performed using a Heraeus CHNO-Rapid VarioEL. X-ray intensity data were collected with a Bruker D8 goniometer equipped with an APEX CCD area detector and an Incoatec microsource (Mo *K*α radiation, λ = 0.71073 Å, multilayer optics). Temperature was maintained using an Oxford Cryostream 700 instrument. Powder diffraction experiments were performed on flat samples at room temperature using a STOE STADI-P diffractometer with Guinier geometry (Cu *K*α, λ = 1.54059 Å, Johann germanium monochromator, STOE image-plate detector IP-PSD and a 0.005° step width in 2θ).

### Refinement

Crystal data, data collection and structure refinement details are summarized in Table 1 ▸. For **1** and **2**, donor H atoms were found in a difference Fourier map. Their positions were refined freely with *U*_iso_(H) = 1.5*U*_eq_(O,N). For **2**, a distance restraint with a value of 0.9 Å was used for atom H1. For **3**, a disordered and a nondisordered structure model were refined. For the major com­ponent, donor H atoms were found in a difference Fourier map and refined with a distance restraint. For the minor com­ponent, donor H atoms were positioned in idealized positions at a distance of 0.85 Å. For the *U*_iso_(H) value of the minority N-bonded H atom in **3**, the *U*_iso_(H) value of the same H atom in the major com­ponent was used and not refined. C-bonded H atoms were positioned geometrically and refined as riding, with C—H = 0.98 Å and *U*_iso_(H) = 1.5*U*_eq_(C).

### Computational details

The single-point calculation was carried out using the program *GAUSSIAN* (Frisch *et al.*, 2016 ▸) with the MIDIX basis set (Easton *et al.*, 1996 ▸). The fragment used was slightly larger than the asymmetric unit to include contacts of symmetry-equivalent residues; this is depicted in the supporting information. The C—H, N—H and O—H distances were corrected to values consistent with results from neutron diffraction experiments (Allen & Bruno, 2010 ▸). The electron density ρ derived from the calculation was then analyzed with *AIMAll* (Keith, 2017 ▸) and *Multiwfn* (Lu & Chen, 2012 ▸), and its topology was described according to Bader’s QTAIM (Bader, 1990 ▸). As suggested by Abramov (1997 ▸), the kinetic energy *G* and *G*/ρ in the bond critical point were derived. Addtionally, the local virial theorem was used to calculate the potential energy *V* (Espinosa *et al.*, 1998 ▸, 1999 ▸).

### Synthesis and crystallization

#### HacaMePz·H*X* (*X* = Cl for 1 and Br for 2)

HacacMePz (20.8 mg, 0.1 mmol) was dissolved in acetone (2 ml). Half-concentrated hydro­halic acid (HCl: 16.6 µl; HBr: 22.8 µl) was then added. Crystals formed approximately 1 h after addition of the acid and the mixture was left unperturbed for slow solvent evaporation. After approximately 80% of the solvent had evaporated, the residual solvent was removed and the sample dried for 30 min *in vacuo*. The product was obtained as rather large colourless block-shaped crystals. Phase purity could be confirmed by powder X-ray diffraction (PXRD).

Hydro­chloride **1**: yield: 14.1 mg (57.6%). CHN analysis calculated (%) for C_11_H_17_ClN_2_O_2_: C 54.0, H 7.0, N 11.5; found: C 54.2, H 7.0, N 11.5.

Hydro­bromide **2**: yield: 19.6 mg (67.8%). CHN analysis calculated (%) for C_11_H_17_BrN_2_O_2_: C 45.7, H 5.9, N 9.7; found: C 45.6, H 5.8, N 9.7.

#### HacacMePz·HBr·0.5TFDIB·H_2_O (3)

HacacMePz (20.8 mg, 0.1 mmol, 2 equiv.) and 1,2,4,5-tetra­fluoro-3,6-di­iodo­benzene (TFDIB; 20.1 mg, 0.05 mmol, 1 equiv.) were each dissolved in CHCl_3_ (2 ml). The two solutions were combined and concentrated hydro­bromic acid (wt% = 48%, 11.4 µl, 0.1 mmol, 2 equiv.) was added. The mixture was left unperturbed for slow solvent evaporation at room temperature. Crystals formed eventually after three weeks. The product was obtained as a colourless crystalline solid (yield: 44.1 mg, 86.8%). Phase purity was confirmed by PXRD. CHN analysis calculated (%) for C_14_H_19_BrF_2_IN_2_O_3_: C 33.1, H 3.8, N 5.5; found: C 34.0, H 3.7, N 5.6.

## Results and discussion

The coordination and crystal chemistry of the heterobifunctional mol­ecule 3-(1,3,5-trimethyl-1*H*-pyrazol-4-yl)acetyl­ace­tone (HacacMePz), which exhibits a β-diketone alongside a Lewis basic pyrazole N-donor atom, was reported recently (van Terwingen *et al.*, 2021*b* ▸). Protonation of the pyrazole N atom is straighforward, but attempts aimed at cocrystallization invariably bear the risk of crystallizing the hydro­halide and/or the XB donor separately. We therefore first address the structures of the hydro­chloride (**1**) and hydro­bromide (**2**) of HacacMePz.

### Crystal structures of HacacMePz·H*X* (*X* = Cl for 1 and Br for 2)

**Figure 1:**
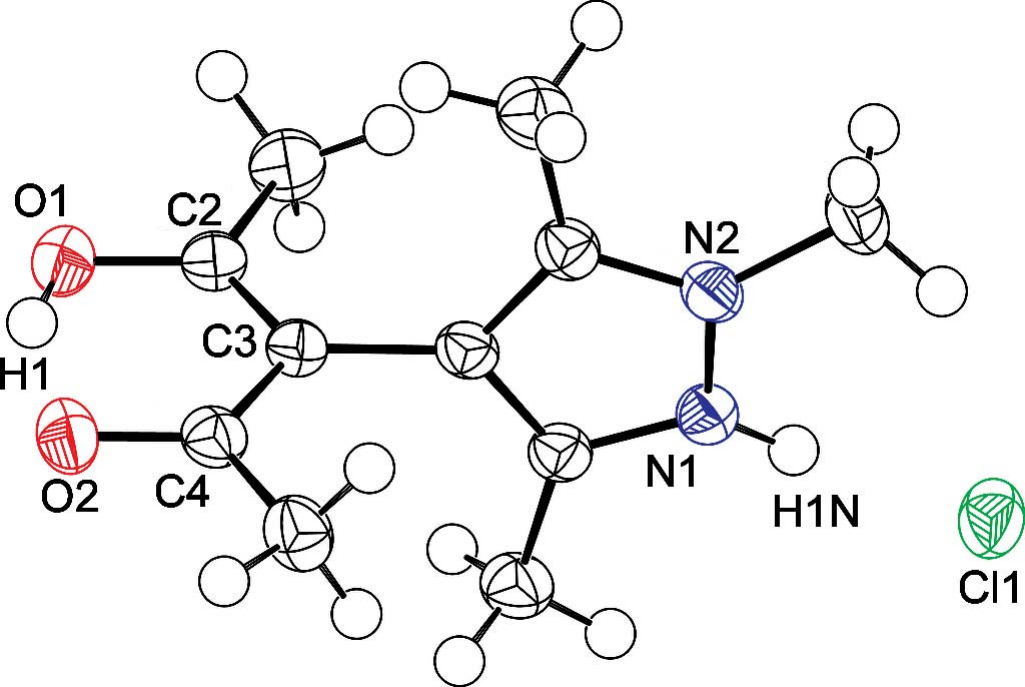
Displacement ellipsoid plot of the asymmetric residue of **1** (80% probability). Selected distances (Å) and angles (°): O1—C2 1.307 (2), O2—C4 1.273 (2), C2—C3 1.391 (2), C3—C4 1.429 (2), N1⋯Cl1 2.9671 (17) and ω 89.12 (9).

The hydro­halides are isostructural; thus, only hydro­chloride **1** will be discussed in detail. We also keep track of the angle ω, which is defined as the angle between the least-squares planes of the β-diketone (atoms O1/O2/C2–C4) and the pyrazole heterocycle (N1/N2/C7–C9). In our previous work, we found this angle to be rather limited to values of approximately 90 ± 17° (van Terwingen *et al.*, 2021*b* ▸). Hydro­chloride **1** crystallizes in the ortho­rhom­bic space group *Pbca*, with *Z* = 8 (Fig. 1 ▸).

**Figure 2:**
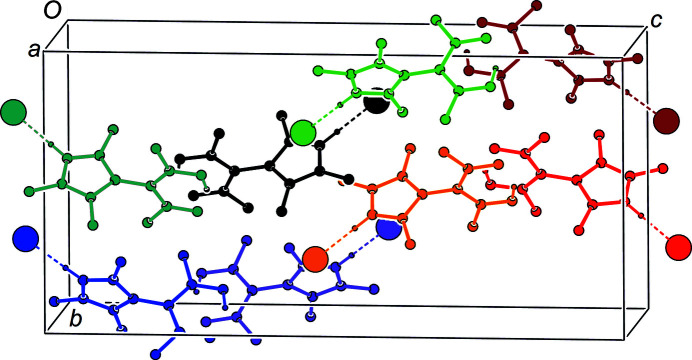
*PLUTON* plot (Spek, 2020 ▸) of the packing in **1**. Different HacacMePz·HCl moieties are depicted in different colours and reveal a classical dipole packing. H atoms not involved in short contacts have been omitted.

As derived from the bond lengths in the acetyl­acetone moiety, the enol H atom is located at O1 forming an intra­molecular hydrogen bond towards O2 with a distance of about 1.6 Å. A closer look at a difference Fourier map before inclusion of the enol H atom into the structure model confirms this suggestion (Fig. S1 in the supporting information); however, a second local maximum of lower electron density at atom O2 can be perceived. Tentative refinement of a structure model with a disordered enol H atom revealed a majority occupation at O1 of 65 (4)%. For the sake of simplicity, we report here the nondisordered model. Positional parameters for both the enol and the pyrazolium H atoms have been freely refined. The pyrazolium H atom forms a hydrogen bond to Cl1, with H⋯Cl = 2.06 (2) Å. The acetyl­acetone and pyrazole groups are nearly orthogonal to each other, with the ω angle being approximately 90°. There are no noteworthy inter­molecular contacts between the HacacMePz·HCl moieties. The closest inter­molecular distances occur between a methyl H atom and Cl1, and amount to approximately 2.7 Å. The arrangement of the hydrogen-bonded ion pairs in **1** corresponds to a classical dipole packing (Fig. 2 ▸).

Comparing hydro­chloride **1** to hydro­bromide **2** reveals only minor differences. Both the *a* and *b* lattice parameter are larger for **2**, while *c* is slightly smaller, resulting in an overall 100 Å^3^ larger unit-cell volume for **2**. The organic residues are almost superimposable, but the hydrogen bond towards the bromide anion is about 0.2 Å longer than that to the chloride anion. Also, the ω angle is slightly less than in **1** at approximately 85°. No sign of enol H-atom disorder could be detected in **2**; this may be due to the unfavourable contrast of the atomic scattering factors in the hydro­bromide com­pared to hydro­chloride **1**. In this context, we also mention the pronounced difference in the linear absorption coefficients of roughly one magnitude (**1**: 0.30; **2**: 3.32 mm^−1^). A synopsis of the important geometric differences between **1** and **2** is given in Table 2 ▸.

### Crystal structure of HacacMePz·HBr·0.5TFDIB·H_2_O (3)

**Figure 3:**
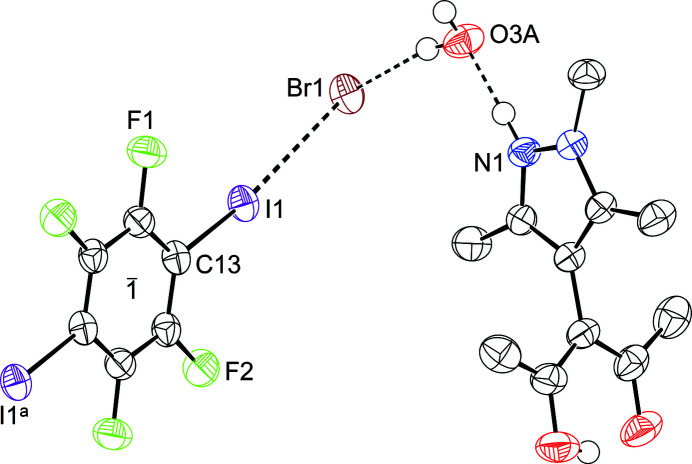
Displacement ellipsoid plot of **3** (80% probability, with C-bonded H atoms omitted). Selected distances (Å) and angles (°): I1⋯Br1 3.2957 (4), Br1⋯O3*A* 3.2843 (16), O3*A*⋯N1 2.641 (2), C13—I1⋯Br1 169.11 (4), I1⋯Br1⋯O3*A* 105.64 (3), Br1⋯O3*A*⋯N1 108.89 (6) and ω 84.49 (9). [Symmetry code: (*a*) −*x* + 1, −*y* + 1, −*z*.]

As mentioned previously, the target cocrystallization and crystallization of the used reactants always com­pete and, thus, are also dependent on the solvent used. This com­petition is not only limited to the reactants (Aakeröy *et al.*, 2013 ▸); Robertson *et al.* (2017 ▸) have shown that hydrogen and halogen bonds also com­pete, and the obtained cocrystal is highly reliant on solvent polarity. We were able to cocrystallize the hydro­bromide of HacacMePz with half an equivalent of TFDIB and one solvent water mol­ecule, and to determine its crystal structure to a high resolution of 1.00 Å^−1^. The com­pound HacacMePz·HBr·0.5TFDIB·H_2_O (**3**) crystallizes in the monoclinic space group *P*2_1_/*c* with *Z* = 4; a displacement ellipsoid plot is shown in Fig. 3 ▸.

Hydrohalide **3** features a hydrogen bond from the pyrazolium H atom towards the cocrystallized water mol­ecule. The water mol­ecule forms hydrogen bonds towards Br1 and its symmetry equivalent Br1^*b*^ [symmetry code: (*b*) −*x* + 2, *y* − 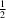 , −*z* + 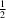 ] with both its H atoms. For Br1 itself, a close contact to I1 in a rather orthogonal fashion regarding O3*A* can be observed: it accepts the σ-hole from I1 and forms a halogen bond. The TFDIB mol­ecule is located on a centre of inversion on Wyckoff position 2*d*. A minor coupled disorder of the *N*-methyl group at N2 of HacacMePz combined with the proton at N1 and cocrystallized water mol­ecule O3 will be discussed later.

**Figure 4:**
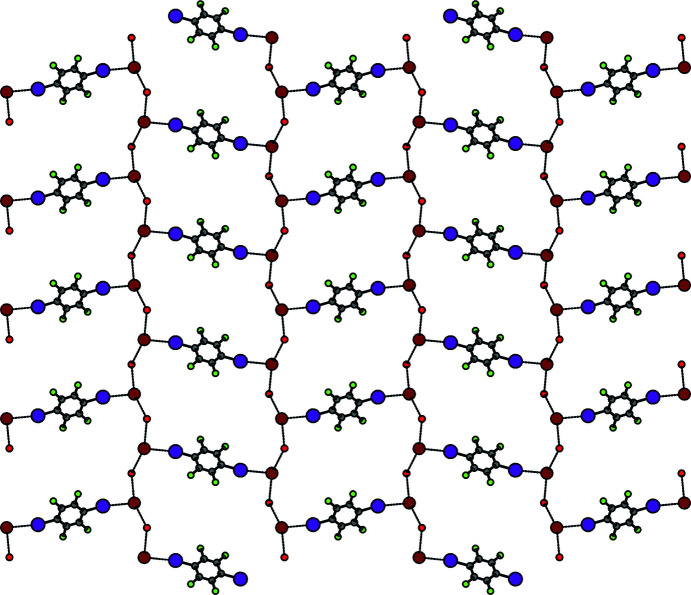
*PLUTON* plot (Spek, 2020 ▸) of the view perpendicular to the (10 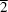 ) plane onto the 2D net in **3** (HacacMePz and H atoms have been omitted).

Expanding the hydrogen-bonded contacts reveals that Br1 accepts two hydrogen bonds from symmetry-equivalent water mol­ecules O3*A*, resulting in a one-dimensional (1D) chain with the graph-set symbol 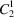 (4) (Etter, 1991 ▸) propagating along [010]. These 1D strands are connected through halogen bonds involving Br1 with the TFDIB mol­ecules, forming a 2D net along the (10 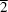 ) plane with meshes formed by 14 vertices. No match for this net could be found in the Reticular Chemistry Structure Resource (RCSR; O’Keeffe *et al.*, 2008 ▸), but if the bromide anions are perceived as three-connected nodes and all other residues as linkers, the topology corresponds to a honeycomb net (**hcb**; Fig. 4 ▸).

After refinement of the structure model, closer inspection of a difference Fourier map revealed two local density maxima in close proximity to the *N*-methyl group (1.79 e^−^) and the protonated pyrazole N1 atom (1.21 e^−^), respectively. The first residual maximum is located at a distance of 1.498 (3) Å from N1 and the second at 2.631 (3) Å from N2. As these distances closely resemble an N—C single bond and an N—H⋯O hydrogen bond, we concluded that the *N*-methyl group is disordered; this disorder is coupled with split positions for the hydrogen-bonded water mol­ecule. The site occupancy refined to 91.1 (4)% for the major component. For the minor water mol­ecule, hydrogen bonds towards two symmetry-equivalent Br1 atoms can be found. They are, however, longer than in the major com­ponent [3.2667 (16) and 3.2843 (16) Å *versus* 3.318 (15) and 3.406 (15) Å], which may contribute to the very different occupancies of approximately 10:1 for the mutually exclusive sites. While at the rather high resolution of 1.00 Å^−1^, the disorder is the most significant residual electron density, truncating the data to 2θ_max_ = 50.3°, higher residuals around Br1 become the most prominent feature of a difference Fourier analysis. Nevertheless, the disorder is apparent also at standard resolution. All geometry data and agreement factors discussed in this article refer to the model described above, which includes the minor disorder. An alternative structure model which does not take atom sites of minor occupancy into account is available in the supporting information.

### Results from a database search

**Figure 5:**
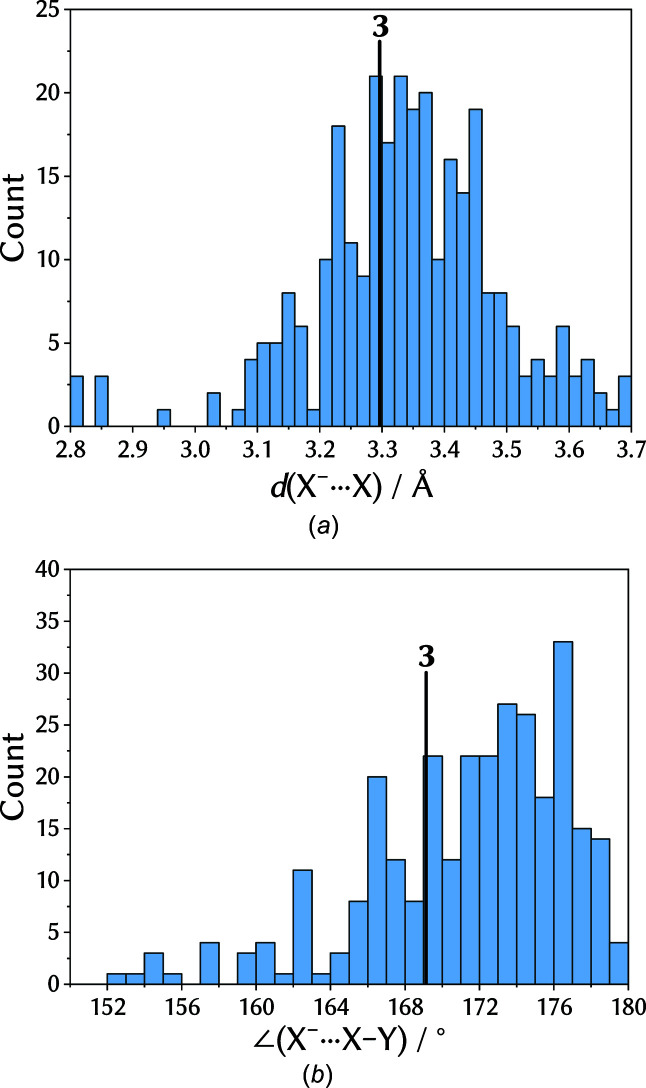
Histograms of the *X* ^−^⋯*X* distance and the *X* ^−^⋯*X*—*Y* angle of the first query run in the CSD, with **3** marked in black.

A search in the Cambridge Structural Database (CSD; Groom *et al.*, 2016 ▸) for similar inter­actions (see supporting information for details) leads to about 200 hits. Analysis of the data shows that the distances and angles for the contacts around the halide in **3** are in the expected range (Fig. 5 ▸). Limiting the search to fluorinated iodo­benzenes reduces the number of matching structures to 12, none of which shows a similar motif to **3**. The closest resemblence is found in OHOVAJ and OHOVIR (Abate *et al.*, 2009 ▸), in which the halogen bonds form a 1D chain which is expanded by amine hydrogen bonds to a 2D net. In our opinion, these results do not necessarily imply that such inter­actions are uncommon; they rather suggest that they have been rarely investigated.

### Electron-density considerations

**Figure 6:**
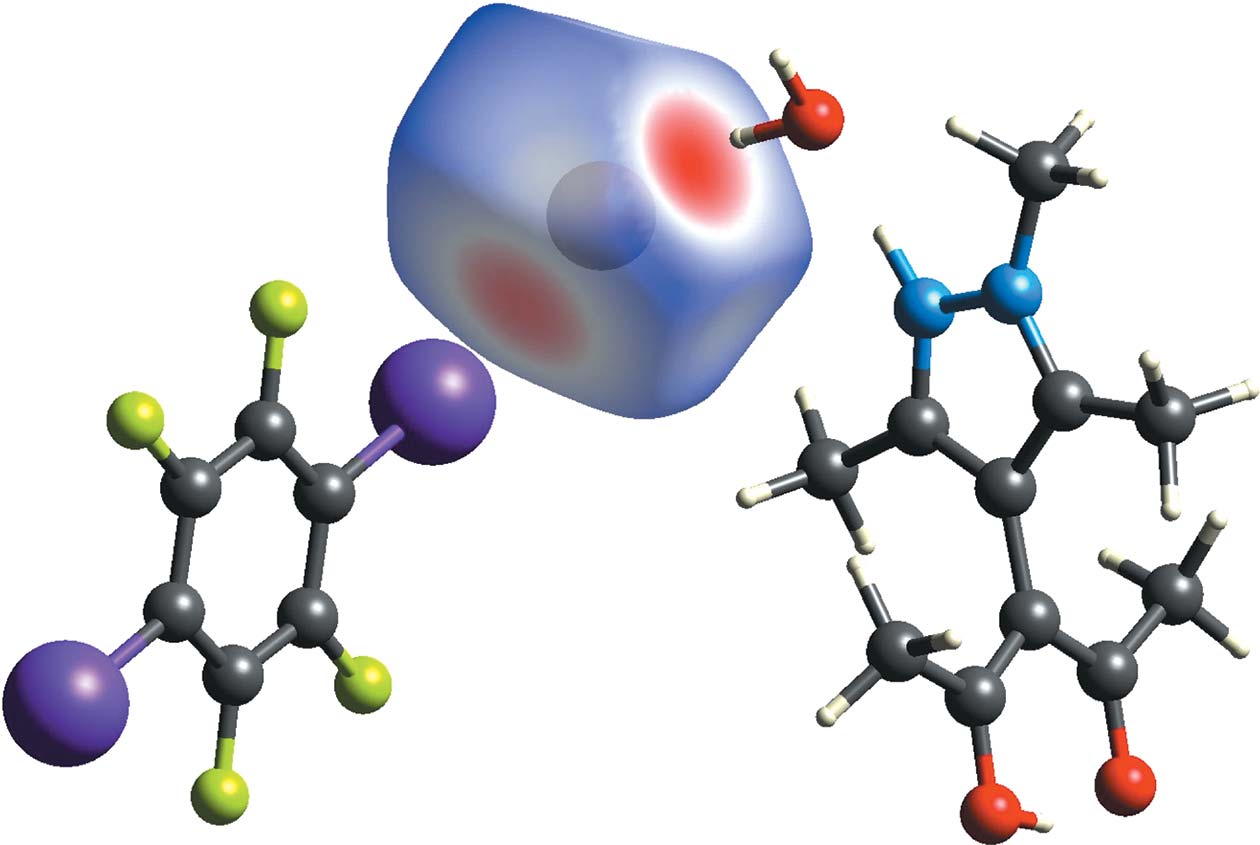
Hirshfeld surface (Spackman *et al.*, 2021 ▸) about Br1 mapped with *d*_norm_; regions in red denote close contacts, while blue denotes long distances.

The Hirshfeld surface (Spackman & Jayatilaka, 2009 ▸) mapped with the distance-sensitive *d*_norm_ criterion reveals close contacts about bromide anion Br1 (Fig. 6 ▸).

**Figure 7:**
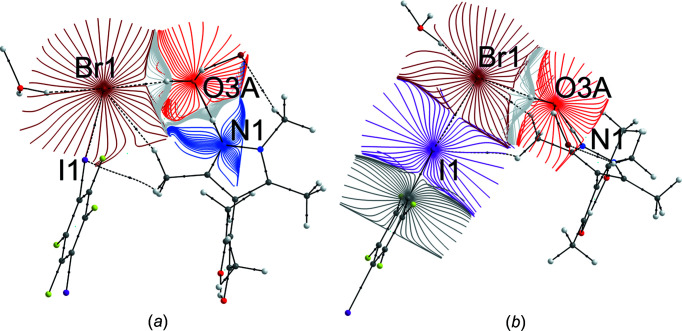
Trajectory plots (Keith, 2017 ▸) of **3**: (*a*) in the N/O/Br plane to reveal hydrogen bonds and (*b*) in the O/Br/I plane for the halogen bond. Intra­molecular and conventionial hydrogen-bond bond paths are shown as full black lines, while the halogen and nonclassical hydrogen bonds are shown as dashed black lines.

In order to gain insight into the electronic situation of **3**, the results of the diffraction experiment were used to calculate the electron density in a single-point calculation; details are provided in the supporting information. The topology of the calculated electron density was analyzed by Bader’s Quantum Theory of Atoms in Mol­ecules (QTAIM) (Bader, 1990 ▸). Covalent bonds and short contacts show up in the gradient of the electron density; two such trajectory plots are shown in Fig. 7 ▸ and show that, in addition to all covalent bonds, both classical N—H⋯O and O—H⋯Br hydrogen bonds exhibit almost linear bond paths and (3,−1) bond critical points (bcps) closer to the H atoms. A linear bond path and a bcp are also encountered for the C—I⋯Br halogen bond.

**Figure 8:**
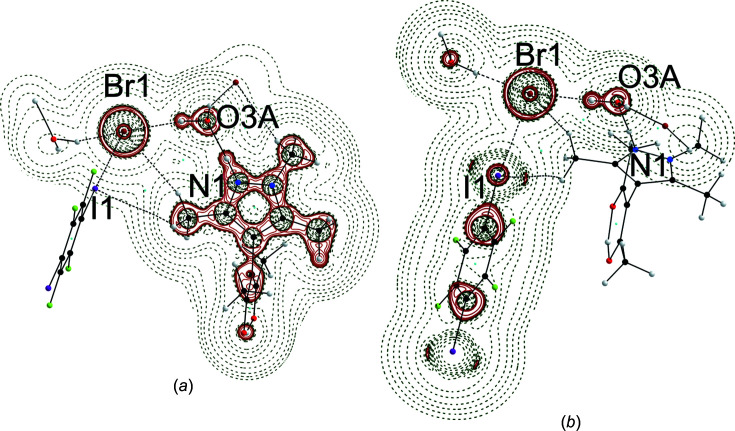
Laplacian of the electron density ρ in **3**; positive values are associated with dark-green dashed lines, while negative values are marked in red. Contour lines are drawn at ±2^*n*^ × 10^−3^ a.u. (0 ≤ *n* ≤ 20).

A synopsis of the relevant data for the electron density in the bcps of the secondary inter­actions is given in Table 3 ▸. For com­parison, a previously published QTAIM analysis of the experimental electron density for an N—H⋯O hydrogen bond (CSD refcode ODEZOO01; Şerb *et al.*, 2011 ▸) has been appended to this table. Despite the similar hydrogen-bond geometry, the N—H⋯O contact in ODEZOO01 does not represent the dominant inter­action; it is associated with significantly lower ρ_bcp_, and the (positive) kinetic energy density *G* and the (negative) potential energy density *V* com­pensate to a negligible total energy density *E*, quite characteristic for weak closed-shell contacts (Espinosa *et al.*, 2002 ▸). The situation is distinctly different in **3**, where the inter­play of *G* and *V* leads to a negative total energy density of −0.02358 a.u., by far the most relevant short contact in **3**. We are not aware of any experimental charge–density studies for O—H⋯Br or Br⋯I inter­actions which might be com­pared to the theoretical results for **3**. Fig. 8 ▸(*a*) shows the Laplacian of the electron density for **3** in the N/O/Br plane. A close look at the N—H⋯O hydrogen bond reveals the oxygen lone pair in the direction of the hydrogen-bond path, but no polarization of the Br^−^ ion towards the water H atom can be perceived. In Fig. 8 ▸(*b*), polarization of the bromide towards the σ-hole of I and the negatively polarized regions on I perpendicular to this Br⋯I contact show up.

**Figure 9:**
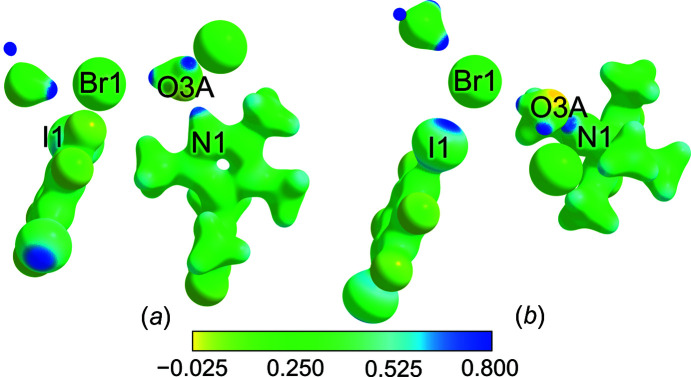
Electrostatical potential for **3** mapped onto the isosurface at an electron-density value of 0.05 a.u. (Keith, 2017 ▸); blue areas mark a positive potential (0.8 a.u.), yellow areas mark a negative potential (−0.025 a.u.) and green areas are associated with a potential of 0.25 a.u.

In Fig. 9 ▸, the electrostatic potential (ESP) has been mapped on an isosurface of electron density; the orientations are the same as in the preceding Fig. 7 ▸. The H atoms attached to O or N atoms are associated with a distinctly positive potential [Fig. 9 ▸(*a*)], and in Fig. 9 ▸(*b*), the positive σ-hole of the TFDIB I atom can be perceived.

## Conclusion and outlook

In this contribution, we have evaluated the hydro­chloride and hydro­bromide of HacacMePz. The successful cocrystallization of the latter with TFDIB supports our earlier claim (van Terwingen *et al.*, 2021*a* ▸) that the combination of hydro­halides with halogen-bond donors is not restricted to a lucky coincidence but is of broader relevance. As expected, the hydrogen and halogen bonds form an angle of roughly 90° around the bromide anion. We want to emphasize the predictability of the spatial arrangement of the two essential and highly directional short contacts. Considering the wide range of available Lewis bases and halogen-bond donors to which this rather underemployed concept may be applied, this combination may offer a new perspective for crystal engineering. We here only mention preliminary results from a closely related experiment; exchanging hydro­bromic acid with hydro­chloric acid leads to a different cocrystal of the com­position HacacMePz·HCl·2TFDIB. In this com­pound, no water and a higher amount of TFDIB is present; the chloride anion accepts one hydrogen bond and three halogen bonds. In contrast to **3**, a 1D halogen-bonded chain is formed, which is not connected by hydrogen bonds in the second dimension. We are currently attempting to design cocrystals of hydro­halides of Lewis bases with XB donors in a rational way and will cover these results in a future contribution.

## Supplementary Material

Crystal structure: contains datablock(s) 1, 2, 3, global. DOI: 10.1107/S2053229622004648/oc3013sup1.cif

Structure factors: contains datablock(s) 1. DOI: 10.1107/S2053229622004648/oc30131sup2.hkl

Structure factors: contains datablock(s) 2. DOI: 10.1107/S2053229622004648/oc30132sup3.hkl

Structure factors: contains datablock(s) 3. DOI: 10.1107/S2053229622004648/oc30133sup4.hkl

Supporting information file. DOI: 10.1107/S2053229622004648/oc30131sup5.mol

Supporting information file. DOI: 10.1107/S2053229622004648/oc30132sup6.mol

Supporting information file. DOI: 10.1107/S2053229622004648/oc30133sup7.mol

Supporting information file. DOI: 10.1107/S2053229622004648/oc30131sup8.cml

Supporting information file. DOI: 10.1107/S2053229622004648/oc30132sup9.cml

CIF for an alternative structure model not taking atom sites of minor occupancy into account. DOI: 10.1107/S2053229622004648/oc3013sup10.txt

Additional information and figures. DOI: 10.1107/S2053229622004648/oc3013sup11.pdf

CCDC references: 2169820, 2169819, 2169818

## Figures and Tables

**Table 1:** Experimental details Experiments were carried out at 100 K with Mo *K*α radiation using a Bruker APEX CCD diffractometer. Absorption was corrected for by multi-scan methods (*SADABS*; Bruker, 2008 ▸). H atoms were treated by a mixture of independent and constrained refinement.

	**1**	**2**	**3**
Crystal data			
Chemical formula	C_11_H_17_N_2_O_2_^+^·Cl^−^	C_11_H_17_N_2_O_2_^+^·Br^−^	C_11_H_17_N_2_O_2_^+^·Br^−^·0.5C_6_F_4_I_2_·H_2_O
*M*_r_	244.71	289.17	508.12
Crystal system, space group	Orthorhombic, *P* *b* *c* *a*	Orthorhombic, *P* *b* *c* *a*	Monoclinic, *P*2_1_/*c*
*a*, *b*, *c* (Å)	8.770 (2), 11.658 (3), 23.843 (6)	8.859 (4), 12.038 (5), 23.805 (10)	12.9151 (2), 11.3061 (2), 12.5239 (2)
α, β, γ (°)	90, 90, 90	90, 90, 90	90, 90.6281 (5), 90
*V* (Å^3^)	2437.9 (11)	2538.7 (18)	1828.62 (5)
*Z*	8	8	4
μ (mm^−1^)	0.30	3.23	3.97
Crystal size (mm)	0.34 × 0.17 × 0.14	0.13 × 0.06 × 0.03	0.19 × 0.15 × 0.08

Data collection			
*T*_min_, *T*_max_	0.619, 0.746	0.595, 0.745	0.563, 0.749
No. of measured, independent and observed [*I* > 2σ(*I*)] reflections	32185, 3104, 2482	26167, 2321, 1647	170884, 15145, 11531
*R*_int_	0.088	0.096	0.091

Refinement			
*R*[*F* ^2^ > 2σ(*F* ^2^)], *wR*(*F* ^2^), *S*	0.042, 0.112, 1.05	0.037, 0.099, 1.04	0.042, 0.093, 1.10
No. of reflections	3104	2321	15145
No. of parameters	156	156	235
No. of restraints	0	1	5
Δρ_max_, Δρ_min_ (e Å^−3^)	0.39, −0.34	0.58, −0.58	1.19, −0.78

Computer programs: *SMART* (Bruker, 2009 ▸), *SAINT* (Bruker, 2009 ▸), *SHELXT2014* (Sheldrick, 2015*a* ▸), *SHELXL2018* (Sheldrick, 2015*b* ▸) and *PLATON* (Spek, 2020 ▸).

**Table 2:** Selected distances (Å) and angles (°) in **1** and **2**.

	O1—C2	O2—C4	N1⋯*X*1	ω
**1**	1.307 (2)	1.273 (2)	2.9671 (17)	89.12 (9)
**2**	1.316 (4)	1.275 (4)	3.159 (4)	85.22 (19)

**Table 3:** Topological properties of important inter­actions of **3** at their bond critical point (3,−1) BPL is bond path length, ρ is the electron density, *G* is the kinetic energy density, *V* the potential energy density and *E* is the total energy density in the bond critical point.

Bond	BPL (Å)	ρ (e Å^−3^)	∇ρ (e Å^−5^)	*G* (a.u.)	*G*/ρ (a.u.)	*V* (a.u.)	*E* (a.u.)
I1⋯Br1	3.2981	0.119	1.11	0.0107	0.61	−0.00988	0.00083
Br1⋯H1O	2.4027	0.158	1.57	0.01684	0.72	−0.01739	−0.00054
Br1⋯O3^i^	3.3112	0.182	1.18	0.01463	0.54	−0.01699	−0.00235
H2O⋯Br1^ii^	2.4257	0.176	1.46	0.01692	0.65	−0.01874	−0.00181
O3⋯H1N	1.7698	0.424	2.1	0.04542	0.72	−0.06900	−0.02358
O⋯H—N^*a*^	1.779 (4)	0.24 (2)	3.69 (3)	0.038	1.07	−0.038	

Note: (*a*) from CSD refcode ODEZOO01 for com­parison (Şerb *et al.*, 2011 ▸). Symmetry codes: (i) −*x* + 2, *y* − 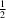 , −*z* + 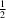 ; (ii) −*x* + 2, *y* + 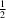 , −*z* + 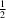 .
