# Supplementary material for: Weaving a 2D net of hydrogen and halogen bonds: cocrystal of a pyrazolium bromide with tetra­fluoro­diiodo­benzene
Source: Acta Crystallogr C Struct Chem. 2022 May 9;78(Pt 6):324–31. doi: 10.1107/S2053229622004648 (PMC9167629; doi:10.1107/S2053229622004648)
Supplement: Supplementary file 11 [file c-78-00324-sup11.pdf]

Supporting Information: Weaving a two-dimensional Net of Hydrogen and Halogen Bonds: Cocrystal of a Pyrazolium Bromide with Tetrafluorodiiodobenzene

Steven van Terwingen,† Ben Ebel,† Ruimin Wang,†,‡ and Ulli Englert*,†,‡

†RWTH Aachen University, Institute of Inorganic Chemistry, Aachen, Germany

‡Shanxi University, Key Laboratory of Materials for Energy Conversion and Storage, Institute of Molecular Science, Taiyuan, Shanxi 030006, People's Republic of China

E-mail: ullrich.englert@ac.rwth-aachen.de

Additional figures

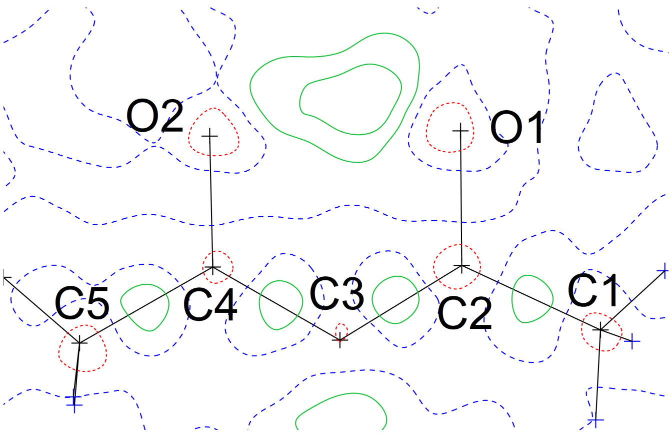

Figure S1: Difference Fourier synthesis map[S1] of **1** before including the enol hydrogen; contour lines drawn at $0.2\,e\,\text{Å}^{-3}$.

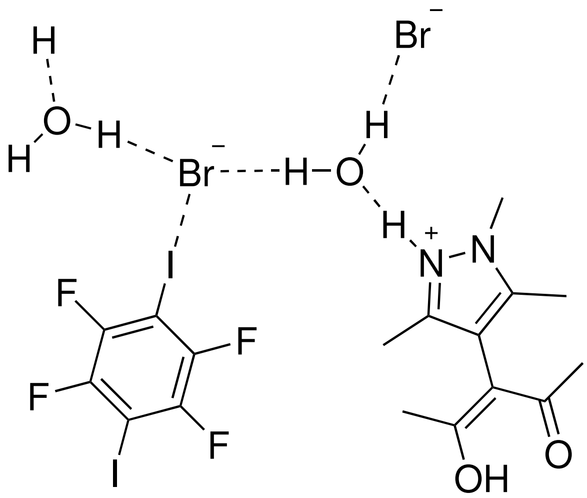

Figure S2: Fragment used for the single point calculation.

Database searches

Database searches in the CCDC[S2] were conducted with ConQuest 2021.2.0, build 327809. The exact structures and constraints are compiled in Figure S3. Van der Waals radii (r_{vdW}) were used as defined in ConQuest.

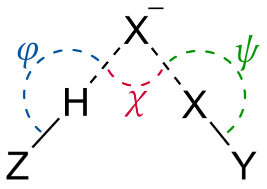

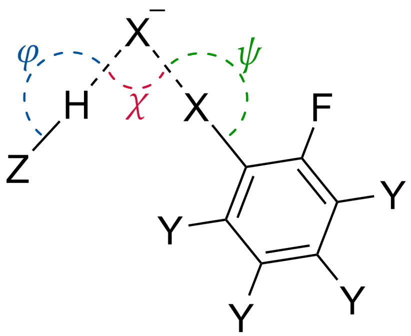

Figure S3: Left: More general query used to classify **3** in the range of related compounds. Right: Query limited to fluorinated halobenzenes as halogen bond donors with only twelve hits. Constraints: $d_{max}(H\cdots X^-) = \sum(r_{vdW})$; $d_{max}(X^-\cdots X) = \sum(r_{vdW}) - 0.2$ Å; $\varphi = 150°$ to $180°$; $\chi = 60°$ to $120°$; $\psi = 150°$ to $180°$; X = F, Cl, Br, I; Y = any atom; Z = O, N.

Powder X-ray diffractograms

Experimental X-ray powder patterns depicted below were collected at room temperature while simulated patterns correspond to the single crystal data obtained at 100 K. This leads to a shift of the lines in the experimental pattern to lower 2θ values due to the lattice expansion at higher temperature. Reflection intensities of **1** only fit the simulated data if the microcrystalline powder is ground; otherwise the intensities suffer from preferred orientation.

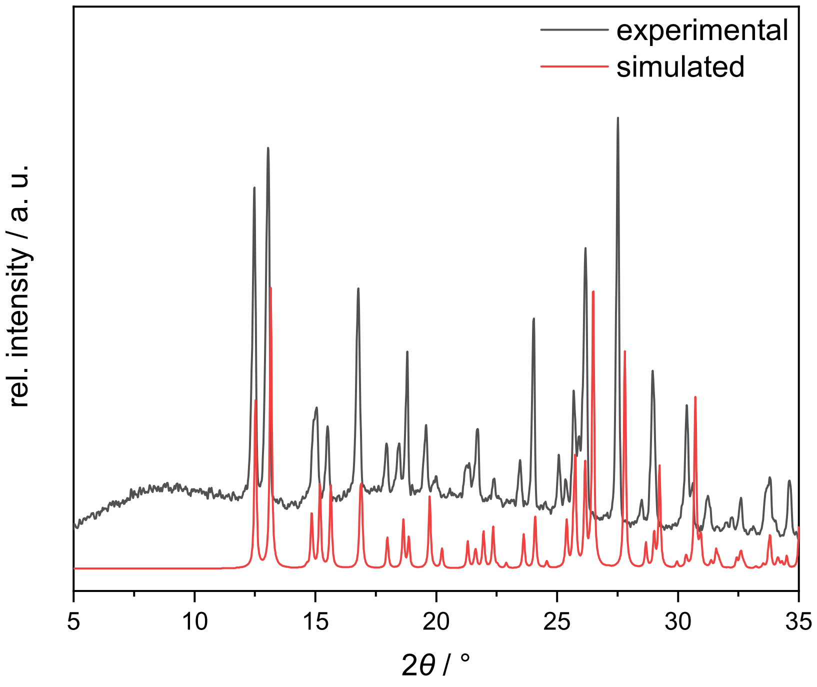

Figure S4: Experimental and simulated powder X-ray diffractogram of **1**.

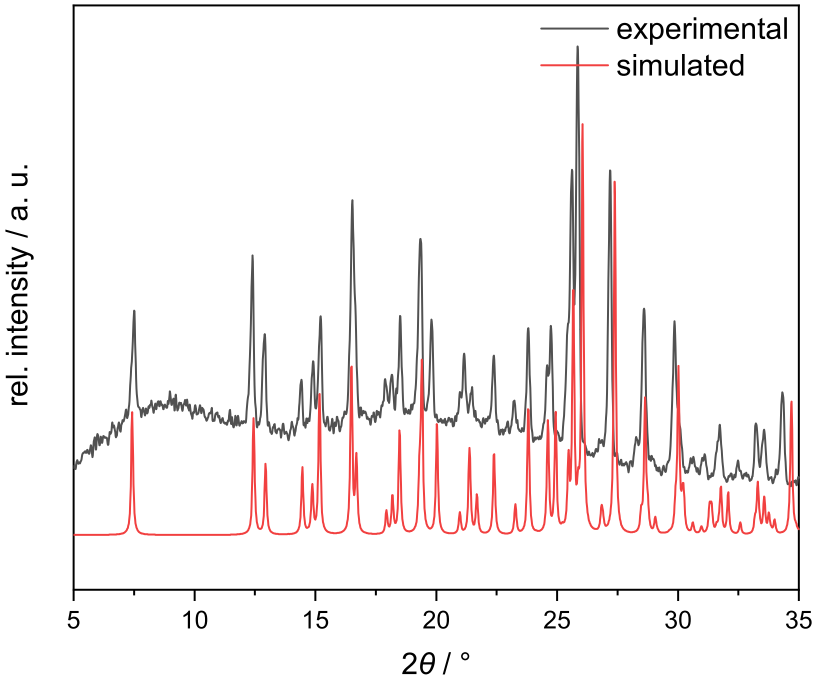

Figure S5: Experimental and simulated powder X-ray diffractogram of **2**.

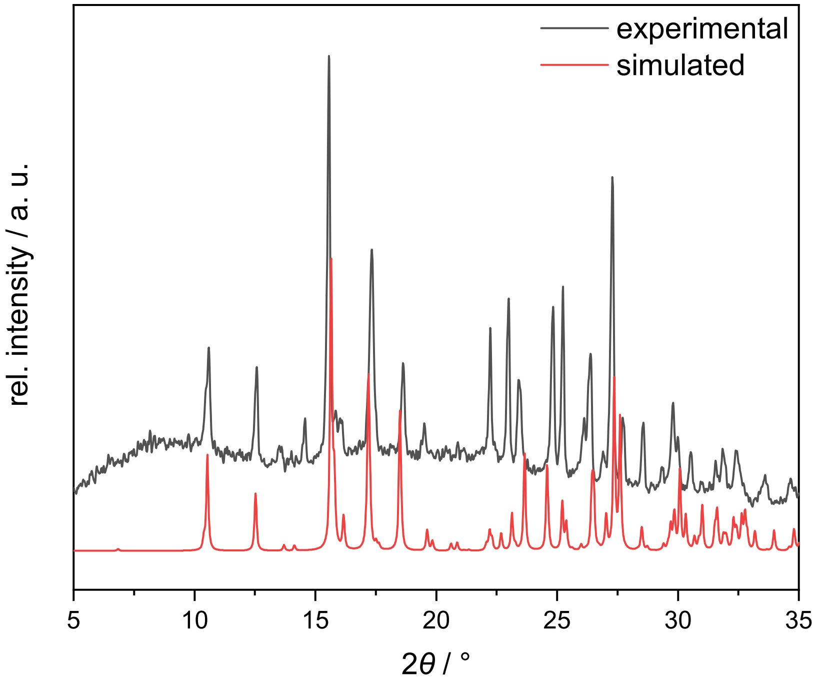

Figure S6: Experimental and simulated powder X-ray diffractogram of **3**.

References

(S1) Spek, A. L. Structure validation in chemical crystallography. *Acta Crystallogr.* **2009**, *D65*, 148–155.

(S2) Groom, C. R.; Bruno, I. J.; Lightfoot, M. P.; Ward, S. C. The Cambridge Structural Database. *Acta Crystallogr.* **2016**, *B72*, 171–179.
